# Whom the Gods Wish to Destroy, They First Make Mad

**Published:** 1913-12

**Authors:** G. Frank Lydston

**Affiliations:** 32 N. State St., Chicago


### Abstracts and Selections.

### Whom the Gods Wish to Destroy, They First Make Mad.

### The Coterie of Politicians Who Run the A. M. A. Finally Shows its Hand.

G. FRANK LYDSTON, M. D.

I present the following to the profession merely as one standing on the side lines watching the passing show. In presenting it I entertain no hope of awakening the physicians of the country to a sense of their danger from medical despotism and trust-monopoly. Many years of hard and expensive endeavor have shown me that the average member of the profession is indifferent to everything save his own individual interests.

Five years ago the political Powers That Be sent from the throne on Dearborn Avenue this message to the profession in answer to my exposé of the putrid conditions that prevailed in medical Denmark:

"There's simply nothing to it. Everybody except Lydston is satisfied with conditions in the A. M. A."

Experience has proved that the oracle of Dearborn Avenue was right. Certain persons were, of course, not satisfied to have certain other persons hold all the offices, otherwise there apparently was no complaint.

I will remark in passing that, in presenting this brief communication, I am not foolish enough to believe that the perusal of it will let the smallest ray of light into the thought chambers of those "insurgents" for place and power only, who consider that the end and aim of reform agitation are only to capture offices for themselves and their satellites. These gentlemen would not recognize a great principle if they met it in the street. Still less would these politicians sacrifice an office to the promulgation of a principle. "Let principles wait on offices," is their motto. Far be it from them to take a political whipping in order to emblazon a principle on the history of medical politics.

And so these gentlemen go empty-handed to State and National Association meetings and empty-handed they return. They simply march up the hill and down again.

"But they get offices." Certainly, but we are discussing reform principles, not political offices, and one set of demagogues is as bad as another.

The profession of Illinois will recall the Surgical Bill which certain parties endeavored to foist on the profession of Illinois. They will remember also, that the moguls of the A. M. A. hastened to disclaim its parentage—which, by the way, was incontrovertibly proved on them. Notice, please, the similarity of the Surgical Bill to the one which I will shortly present. That the two bills were the fruit of the same political loins is self-evident.

I have elsewhere asserted that the efforts of the officials in control of the A. M. A. to secure a Federal Bureau of Health were not sincere. I reiterate here, what I have before publicly stated, viz.: *The Medical Trust cares not a continental for the best interests of the profession and the people of the United States. It merely wants an absolute monopoly of all things medical in this country.*

A. M. A. officialdom is howling in the open for a Federal Bureau of Health. Under the rose it has combined with the medical departments of the government—notably with the Marine Hospital Service—to knife said Bureau of Health.

Noting that the members of the National Bureau of Health Committee which they had appointed was likely to rise above its source, the A. M. A. machine crowd proceeded to throw mud at said committee—as witness what happened at the last A. M. A. "round-up" in Minneapolis. A prominent surgeon on the committee was openly accused by the machine of soliciting funds for bribery at Washington.

*Fearing that their "pot vs. kettle" tactics would fail, the A. M. A. gang next proceeded to attempt to get a "cinch" on the only thing which a Federal Bureau of Health would bring them that they cared a hang about, viz.: absolute control of the teaching, licensing and practice of medicine in the U. S.!*

The bill which I here present shows how the gang set about accomplishing its fell design.

63rd Congress—First Session.

H. R. 8606.

In the House of Representatives. (Sept. 27, 1913.)

Mr. Reilly of Connecticut (by request) introduced the following bill, which was referred to the Committee on Military Affairs and ordered printed.

A BILL TO CREATE A UNITED STATES MEDICAL LICENSING BOARD.

Be it enacted by the Senate and House of Representatives of the United States of America in Congress assembled; That the President be, and he is hereby, authorized and directed to appoint *two medical officers of the United States Army,* with the rank of captain and of major; *two medical officers of the United States Navy,* with rank of Lieutenant and Lieutenant Commander; and *two medical officers of the United States Marine Hospital Corps,* with the rank of Lieutenant and Lieutenant Commander, to a board to be known as the *United States Medical Licensing Board.*

Sec. 2. That the terms of the members of the board to be four years each, and the salary of each member thereof to be $4,000 per annum with mileage. Said board shall be in continuous session at Washington when not on duty in various States.

Sec. 3. That all regular licensed medical practitioners of medicine, now holders of a medical diploma and a State license permitting them to practice in the respective States, shall upon the passage of this act by presenting to said Board their medical diploma, their State medical license, and any other diplomas they may have, and upon the payment of the sum of $2 be given a United States License which will permit them to practice their profession of medicine in any State or Territory of the United States and its possessions.

Sec. 4. That the United States Licensing Board shall hold its meetings in various cities of the United States and shall examine all newly-graduated medical doctors so that they may obtain a United States license, which license will permit them to practice medicine or surgery in any State or Territory of the United States and its possessions without any further examination: Provided,—That any candidate for said license shall fulfill all the requirements of *the American Medical Association and shall be an American citizen* and present a high school certificate or its equivalent and shall have a doctor of medicine diploma from *a medical college in good standing, as declared by the American Medical Association,* and upon the payment of $10 and the filing of certificates of good moral character shall be admitted to examination and upon the passage of said examination shall be granted a United States License, which will permit the holder to practice medicine and surgery in any State or Territory of the United States and its possessions.

Sec. 5. That the license may be revoked in case abortions or other *unprofessional conduct* and criminal acts are performed.

The profession will please observe:

1. That there is nothing subtle about this bill. On the contrary it is as ingenuous as it is asinine and devoid of the slightest knowledge of constitutional law.

2. That a foreign-born physician would have to wait several years for his naturalization papers before he could apply for an examination for a "United States" license. He could not then qualify unless his Alma Mater were approved by the A. M. A. The "Cock Robins" of befo' de war, could not have been less liberal.

I reverently invoke the shade of Christian Fenger to pass judgment on this insult to the memories of certain great men. I might appeal to the great men of foreign birth and education who are still with us and already licensed to practice medicine, were I sure that they care what may happen to those who come after them. As for those medical foreigners who are feeding at the A. M. A. trough, they probably are very well satisfied.

3. If this bill should pass,—which of course, it can not, unless Congress is merely an aggregation of imbeciles from the idiotic actions of which the Supreme Court alone can protect our suffering country—the A. M. A. need no longer classify medical colleges. It can arbitrarily settle the fate of all schools save those run by its own satellites.

4. It is proposed that the medical despotism under which we now are struggling to survive, shall become *a medico-military despotism!* Imagine the profession under the dominance of the A. M. A.—military combine!

(I have already publicly shown how the A. M. A. gang and the military medical authorities have combined for their mutual interests and against the best interests of the profession at large. I have in my possession evidence which apparently shows that the military medical authorities and the A. M. A. gang have combined in at least one important matter to pervert scientific truth for reasons best known to themselves.)

5. Note please, that $2 per capita will mulct the physicians now practicing in the U. S. to the tune of something like $300,000! They surely will all want licenses, and the next step probably will be a compulsory law. As Mr. Dooley said, "That's wan way of gittin' the money."

6. Note, please, the enormous revenue to be derived from "newly graduated medical doctors" at $10 per.

Will the various States stand passively by and see their own constitutional rights abrogated and their little graft raped from them? Perish the thought—and the national robbers!

7. Note, please, what will happen under the bill to anybody who is guilty of "unprofessional conduct." *Remember that the A. M. A. will put the stamp on our conduct.* The Lord help an outsider who gets a full-page syndicate unpaid newspaper ad! If he doesn't happen to be a member of the A. M. A. or a Fellow of the Royal American College of Surgeons—to the Bastile with him!

The foregoing is merely by way of emphasis and substantiation of what I have elsewhere and often publicly contended regarding the personnel and policies of the A. M. A. Personally, I think it would be a good thing for the profession if such a bill as that under consideration were "put over." The doctor might wake up and show that he does not like the pressure of the foot on his neck so well as he now appears to do. But then, it might be too late.

N. B.—Since the foregoing was written the Appellate Court of Cook county, Illinois, has handed down a decision (Oct. 7, 1913) sustaining my contentions regarding the operations of the American Medical Association.

1. That the elections of the American Medical Association cannot legally be held outside the State of Illinois.
2. That the membership at large should have a direct vote.
3. That non-members should not be privileged to vote.
4. That the delegate system is illegal.

A. M. A. officialdom moved heaven and earth to prevent by hook or crook a legal decision on the matters at issue. The late John E. W. Wayman, States Attorney, complaisantly refused to serve quo warranto writs on the trustees of the A. M. A. All sorts of technical quibbles and worse methods of evading the issue were indulged in by the A. M. A. officials, but without avail. I have succeeded in getting a decision which I feel confident will be sustained by the Supreme Court, inasmuch as my contentions were based upon Supreme Court decisions. *Should the Supreme Court sustain the lower court, I will have the pleasure of presenting on a platter to the membership of the American Medical Association at the next meeting the ownership of the Association, of which a self-seeking, arrogant oligarchy robbed us nearly fifteen years ago at St. Paul.*

It has taken three years and a half to win a decision against as selfish a gang of medical politicians as ever feared to submit a doubtful cause to a fair and impartial hearing. At present writing the courts have decided that *the operations of the American Medical Association for nearly fifteen years have been illegal.*

The contentions above mentioned were embodied in the resolutions offered by me at the Danville meeting of the Illinois State Society and opposed by the "gang," as well as by certain alleged "insurgent" leaders.

32 N. State St., Chicago.

---

Glass and tube drains should never be allowed to rest against a large bloodvessel (*e. g.*, the epigastric, the internal iliac). They may cause fatal erosion.—*American Journal of Surgery.*

